# Prevalence of needle-stick injury among nursing students: A systematic review and meta-analysis

**DOI:** 10.3389/fpubh.2022.937887

**Published:** 2022-08-15

**Authors:** Xu Xu, Yu Yin, Hao Wang, Fengxia Wang

**Affiliations:** ^1^Department of Emergency, Affiliated Hospital of North Sichuan Medical College, Nanchong, China; ^2^Disinfection Supply Center, The Second Affiliated Hospital of Chongqing Medical University, Chongqing, China

**Keywords:** needle-stick injury, nursing student, not reporting needle-stick injury, meta-analysis, sharp injury

## Abstract

**Introduction:**

Needle-stick injuries (NSI) are a serious threat to the health of healthcare workers, nurses, and nursing students, as they can expose them to infectious diseases. Different prevalence rates have been reported for this type of injury in different studies worldwide. Therefore, this study aimedto estimate the pooled prevalence of NSI among nursing students.

**Methods:**

This study was conducted by searching for articles in Web of Science, PubMed, Scopus, Embase, and Google Scholar without time limitation using the following keywords: needle-stick, needle stick, sharp injury, and nursing student. The data were analyzed using the meta-analysis method and random-effects model. The quality of the articles was evaluated with Newcastle-Ottawa Quality Assessment Scale (NOS). The heterogeneity of the studies was examined using the *I*^2^ index, and the collected data were analyzed using the STATA Software Version 16.

**Results:**

Initially, 1,134 articles were retrieved, of which 32 qualified articles were included in the analysis. Nursing students reported 35% of NSI (95% CI: 28–43%) and 63% (95% CI: 51–74%) did not report their needle-stick injuries. The highest prevalence was related to studies conducted in Asia (39.7%; 95% CI: 31.7–47.7%). There was no significant correlation among NSI prevalence and age of samples, and article year of publication.

**Conclusion:**

A third of nursing students reported experiencing NSI. Consequently, occupational hazard prevention training and student support measures need to be considered.

## Introduction

An injury caused by a needle or a sharp object is known as a needle-stick injury (NSI). The injury is usually not serious, so this does not pose a serious risk, but it may pose problems if the needle is contaminated with blood or other body fluids (1). The risk of a single exposure to virus-infected blood for hepatitis B, hepatitis C, and Human Immunodeficiency Virus (HIV) is 6–30%, B, 0–7%, and 0.2–0.5%, respectively (2). Around 80,000 infections per year were estimated to occur in healthcare staff globally in 2,000 due to lack of intervention to prevent NSI (3). Health care staff were estimated to contract 80,000 infections per year as a result of NSIs not being prevented in 2000 (4, 5). Healthcare workers can suffer mental distress, anxiety, depression, and post-traumatic stress disorder due to NSI, leading to more work absences and lost workdays (3, 5–8). These injuries are more common in nurses and nursing students than in other healthcare workers (9, 10). The majority of nursing students' training takes place in clinical settings, where they learn various nursing skills, including injection techniques, taking blood samples, and monitoring blood sugar levels using glucometers under the supervision of instructors. However, these nurses more vulnerable to NSI than experienced nurses due to inadequate knowledge and experience in terms of handling needles and sharp objects in a clinical setting (11).

Many studies have investigated NSI among health professionals, but students have often been neglected. Thus, NSI prevalence should be investigated among students. A recent systematic review and meta-analysis focused only on nursing interns, and few studies (eight papers) were retrieved and analyzed due to the limitation in the target population and searched databases (12). In addition, it is impossible to put them all in one group and estimate the pooled prevalence of NSI because the nature of the nursing students' work is different from that of students in other medical fields. Therefore, this study focused on nursing students. Previous studies worldwide have estimated the prevalence of NSI in nursing students and reported different results. Based on the literature review, the prevalence of NSI among nursing students has varied between 8.7 and 71% (13, 14). Our first step in preventing this problem is to gain an understanding of its exact prevalence. Therefore, this study aimedto estimate the pooled prevalence of NSI in nursing students all over the world.

## Methods

### Search strategy

The present systematic review and meta-analysis were conducted according to Preferred Reporting Items for Systematic Reviews and Meta-Analyses (PRISMA) guidelines. The search was conducted in databases of Web of Science, PubMed, Scopus, and Google Scholar without time limitation using the following keywords: needle-stick, needle stick, sharp injury, nursing student, and their possible combinations. The discussion section and reference list of each article was reviewed for more access to articles. The search strategy in the PubMed database was as follows: (“Needlestick Injuries” [Mesh] OR “Needle?stick Injur^*^”[tiab] OR “Needle?stick^*^”[tiab] OR “Sharps Injur^*^”[tiab]) AND (“Students, Nursing”[Mesh] OR “Nursing Student^*^”[tiab] OR “Pupil Nurse^*^”[tiab] OR “Nursing Staff^*^”[tiab]).

### Study selection and data extraction

The study included all observational studies published in English that examined NSI among nursing students. Nursing students' data were analyzed when available for studies conducted on diverse groups of healthcare workers; otherwise, they were excluded from the analysis. In addition, interventional, review, and qualitative studies, as well as editor letters, and conference papers were excluded. Two independent authors selected eligible articles by reviewing their titles and abstracts. Article information, including first author, publication year, the mean age of samples, sample size, place of study, number of students with NSI, and number of students not reporting their injury, was extracted. Disagreements between the authors were resolved through discussion.

### Quality assessment

Methodological quality was evaluated for the selected articles to minimize bias. For this purpose, the Newcastle-Ottawa Quality Assessment Scale (NOS) was used to evaluate each study using six items in three groups, including selection, exposure, and comparability with the maximum score as much as 9. The opinion of the corresponding author was applied in case of disagreement in scoring the selected articles (15).

### Data analysis

Heterogeneity across studies was examined using Cochran's Q test (*p* < 0.1) and *I*^2^ statistics. According to *I*^2^ statistic, heterogeneity was divided into three categories of below 25% (low heterogeneity), between 25 and 75% (medium heterogeneity), and over 75% (high heterogeneity) (16). The random effects model was used to estimate the pooled prevalence of NSI with a 95% confidence interval. The pooled prevalence of NSI was estimated using the random effects model due to a high heterogeneity across the studies, and the heterogeneity was 99.10%. This type of meta-analysis is limited by high heterogeneity, which is inevitable in meta-analyses that aim to estimate pooled prevalence (17). The subgroup analysis was performed by continent (Asia, Europe, and others) and work time (NSI prevalence in the last year, NSI prevalence during the internship, and unknown) to identify potential sources of heterogeneity. NSI prevalence was examined using univariate meta-regression analysis based on the mean age of samples and the year of publication of the article. Publication error was visually analyzed by examining funnel plots using Egger's method. Statistically significant results were only included in the analysis because publication bias was present, and negative or non-significant results were excluded because they had not been published (18). All the statistical analyses were performed using Stata Software, Version 16.

## Results

**Figure 1:**
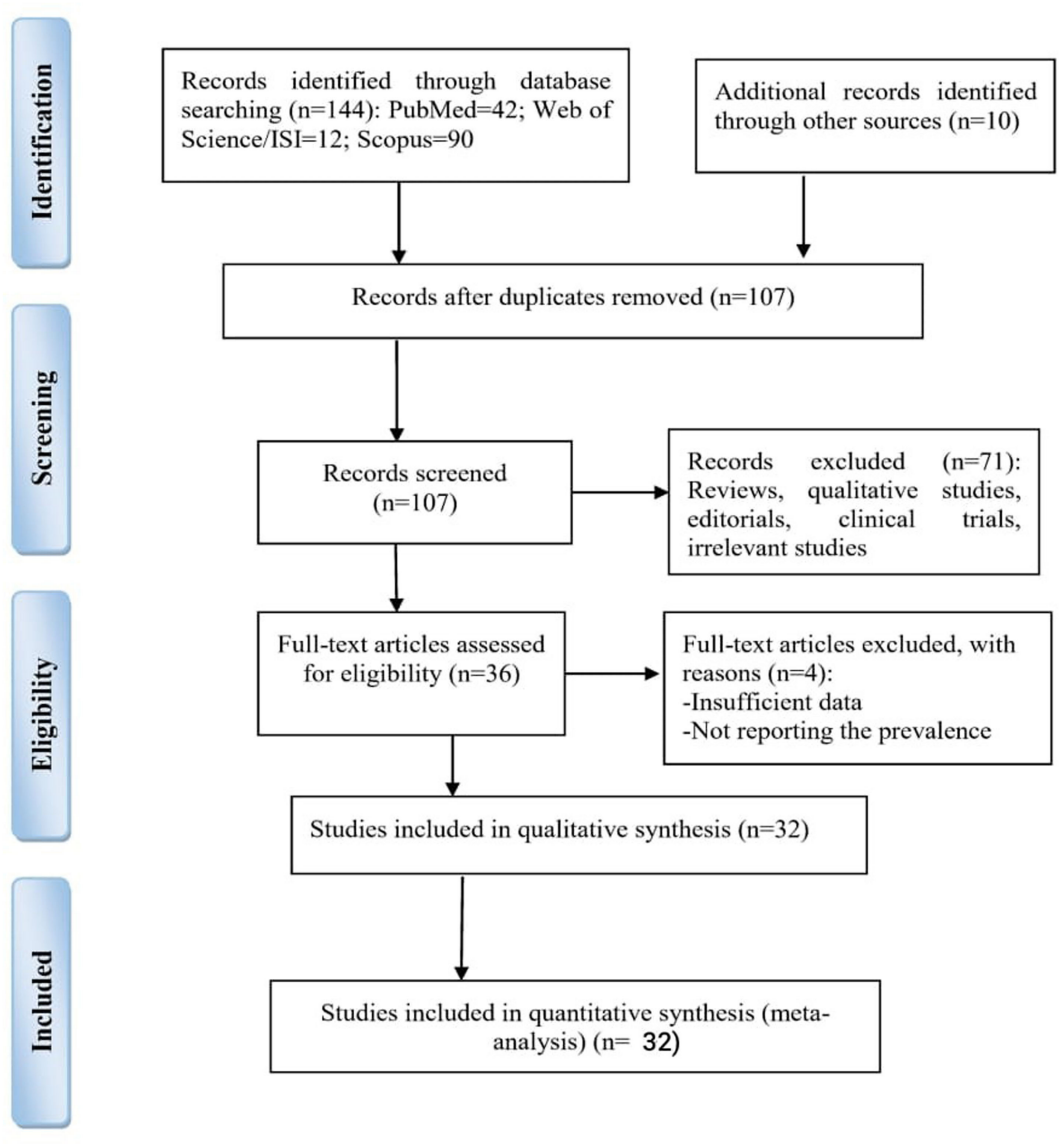
Flow diagram of the search and screening processes based on PRISMA 2009.

A total of 1,134 articles were found in the initial search, of which 516 duplicate articles were excluded. In the next step, the titles and abstracts of the remaining articles (*n* = 618) were reviewed, and the qualified articles were included in the analysis based on the inclusion criteria (Figure 1).

A total of 32 studies with a sample size of 11,622 were included in the analysis. There were 13 studies that did not report NSI. Most studies were conducted in Turkey, China, Iran, and India (every four studies), respectively. The large stand lowest sample sizes were related to Veronesi et al. (5) (*n* = 2,742) and Bathija et al. (19) (*n* = 58), respectively (Table 1).

**Table 1:** Characteristic of the included articles.

**References**	**Sample size**	**Country**	**Prevalence of NSIs (%)**	**Prevalence of under-reporting (25)**
Mishra et al. (20)	312	India	25	
Al Qadire et al. (21)	167	Oman	18.2	-
Wang (22)	400	China	67	
Ledinski Fičko et al. (23)	149	Croatia	10.7	
Zagade et al. (24)	250	India	23.3	17.2
Bagnasco et al. (25)	238	Italy	39	-
Ditching et al. (26)	233	Philippines	15	54.2
Nawafleh et al. (27)	162	Jordan	46	43
Suliman et al. (28)	279	Jordan	26.3	67.1
Veronesi et al. (5)	2742	Italy	11.5	-
Zhang et al. (29)	393	China	60.3	86.9
Prasuna et al. (30)	83	India	39.7	54.5
Hosseini Senjedak et al. (31)	214	Iran	56.3	-
Bathija et al. (19)	58	India	44.8	-
Cheung et al. (14)	878	Hong Kong	8.7	61
Ozer and Bektas (32)	258	Turkey	33.3	-
Unver et al. (33)	218	Turkey	47.2	13.5
Lukianskyte et al. (34)	100	Lithuania	78	92
Small et al. (35)	197	Namibia	17	55
Baghcheghi et al. (36)	227	Iran	43	
Baghcheghi et al. (36)	227	Iran	70	
Irmak and Baybuga (37)	310	Turkey	19.3	68.3
Yao et al. (38)	246	China	26	96.2
Hulme (39)	79	Uganda	25.3	-
Talas (40)	473	Turkey	48.8	55.8
Zhang et al. (2)	213	China	12.2	-
Blackwell et al. (41)	96	USA	9.4	95.8
Massaro et al. (42)	223	Italy	17.9	-
Askarian and Malekmakan (13)	688	Iran	71	82
Smith and Leggat (43)	274	Australia	13.8	39.4
Yang et al. (44)	527	Taiwan	50	60.9
Shiao et al. (45)	708	Taiwan	61.8	69.8

**Figure 2:**
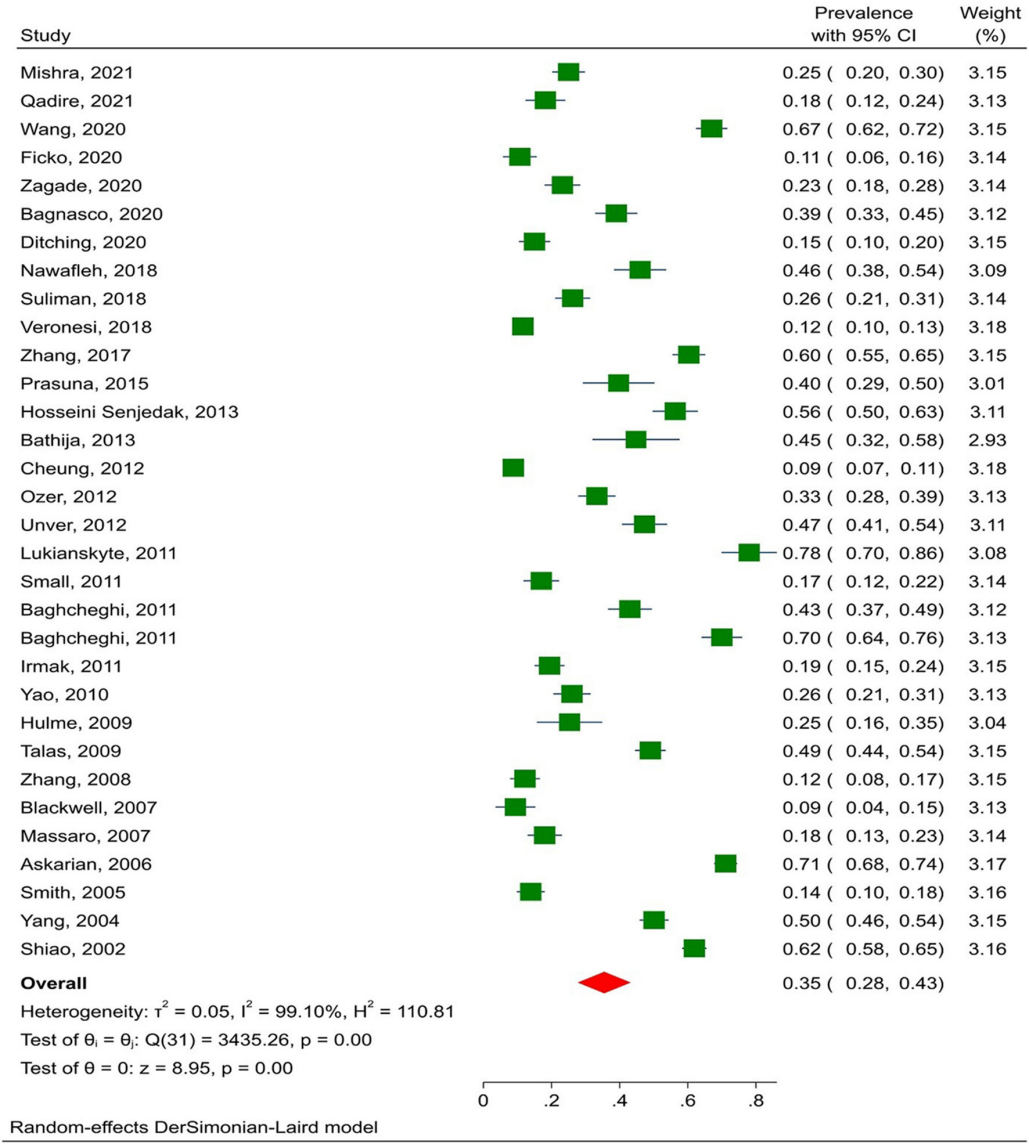
The prevalence of NSI among nursing students.

The prevalence of NSI among nursing students was assessed for publication bias to ensure that all relevant studies were included in the analysis. The results showed that publication bias was significant (*p* = 0.169), and the pooled prevalence of NSI in nursing students was 35% (95% CI: 28–43%) (*I*^2^ = 98.9%, *p* = 0.001) (Figure 2). In addition, the subgroup analysis by continent and injury time showed that the highest prevalence rates of NSI were related to Asia (39.7% with 95% CI: 31.7–47.7%) and internship period (37.6%; 95% CI: 26.6–48.6%). In some studies, the injury time was not specified, and the injury time was broken down into last year or the internship period. The prevalence of NSI was higher in the internship period than in other periods (*p* = 0.888; Table 2). Meta-regression analysis found no relationship between NSI prevalence and article publication year, sample size, or mean age of students.

**Table 2:** The subgroup analysis by period and continent.

**Subgroup**	***N***	**Prevalence (95% CI)**	**Between studies**			**Between subgroups**	
			***I* ^2^**	***P***	***Q***	***Q***	***P***
**Work time**
During internship	12	37.6% (26.6–48.6%)	98.97%	0.001	1,946.68	0.24	0.888
Last year	3	34.3% (24.7–44%)	82.6%	0.001	10.18		
Unknown	17	34.2% (22.8–45.7%)	99%	0.001	1,446.18		
**Continents**
Asia	23	39.7% (31.7–47.7%)	98.5%	0.001	2,293.98	24.48	0.001
Europe	5	31.3% (6.5–56.1%)	99.4%	0.001	320.74		
Other	4	15.5% (10.1–42.7%)	71.28%	0.028	8.74		

**Figure 3:**
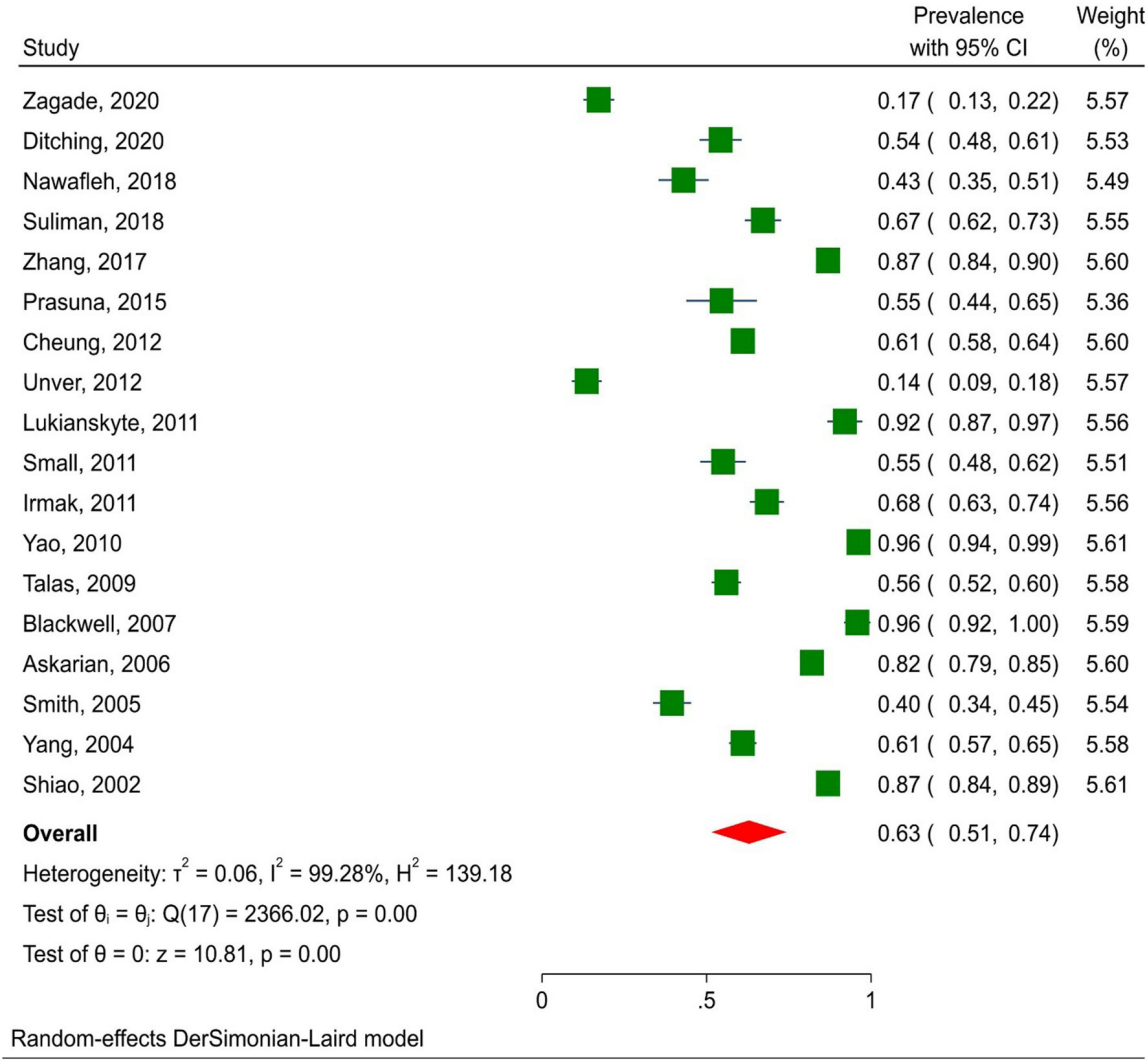
The frequency of NSI non-reporting among nursing students.

According to the results, the prevalence of not-reported NSI among nursing students was 63% (95% CI: 51–74%) (Figure 3). Asian studies reported NSIs at a lower rate than those on other continents (60.7% vs. 63.5%, *p* < 0.01). Furthermore, the frequency of non-reporting of injury was 56.4% during internship and 69.4% in other periods (*p* = 0.256). There was no significant correlation between NSI prevalence, age, and publication year of articles.

## Discussion

This study aimed to estimate the pooled prevalence of NSI in nursing students worldwide. The results showed that 35% of nursing students had experienced NSI. Bouya et al. (46) analyzed 11 studies and found a prevalence rate of 45.3% for NSI among nursing students, which was a higher prevalence rate than in the present study. The studies were conducted during different periods, which can explain the difference. The results of another meta-analysis in China showed that the prevalence of NSI in nursing students was 33%, which is consistent with those of the present study (47). A recent systematic review and meta-analysis investigated occupational injuries among nursing interns and found that the prevalence of NSI in this group was 27%, which is lower than the present study (12). The previous systematic review and meta-analysis analyzed eight studies, two of which were semi-experimental. Bringing these studies together with observational studies to estimate the pooled prevalence is methodologically incorrect because the nature of these studies is different. There were also three studies performed on medical students (not nursing students) and one retrospective study. The remaining two studies were included in our analysis. In addition, Scopus (the world's largest database) and Web of Science/ISI were not searched. The present study resolved the limitations mentioned above, and covered the NSI in all nursing students by searching all four primary databases (without a time limit).

The literature review indicated that most previous studies were focused on the prevalence of NSI in healthcare professionals, and examining this problem in nursing students had primarily been ignored by previous studies. Infectious diseases such as hepatitis and AIDS can be spread through NSI in students, preventing them from obtaining future employment opportunities. A previous study showed that half of the healthcare workers had an NSI during their work time, and one-third had it the previous year (48). Nursing students appear more vulnerable to NSI than healthcare professionals due to inadequate knowledge and experience.

The study results related to the continent revealed that the prevalence of NSI among nursing students was higher in Asia than in other continents. Bouya et al. (46) found that the highest prevalence rates had been reported in the studies conducted in Asian countries regarding exploring the prevalence of NSI in healthcare professionals. Although different prevalence rates of NSI are reported in different regions, the high prevalence of this problem in Asian countries may be related to different study designs, sample sizes, and national and local prevention policies.

This study showed that 62.9% of nursing students suffering from NSI did not report their injuries. Students deprive themselves of timely medical examination and receiving prophylaxis and examination of early changes in serum antibodies immediately after exposure by not reporting their NSI (49), which may transmit the potential viruses to their family members (50, 51). Not reporting NSI is a significant clinical challenge that may have undermined the validity of the existing data regarding this problem (52). Some of the most important reasons for not reporting NSI include stigma, lack of awareness (53), negative career consequences, shame (54), fear of being blamed, fear of creating more problems (55), thinking that NSI is none of others' business, and believing that reporting the injury would not be helpful (56).

Overall, the study results indicated that NSI is widespread among nursing students and that most do not report their injuries. NSI and its negative consequences can be reduced by holding workshops regarding workplace safety, providing more support for nursing students in clinical settings, and encouraging students to report their injuries.

## Data availability statement

The original contributions presented in the study are included in the article/supplementary material, further inquiries can be directed to the corresponding author.

## Author contributions

Concept and design: XX and FW. Acquisition, analysis, or interpretation of data: YY. Drafting of the manuscript: YY and XX. Critical revision of the manuscript for important intellectual content: HW. Statistical analysis: FW. All authors gave their final approval of this version of the manuscript.

## Conflict of interest

The authors declare that the research was conducted in the absence of any commercial or financial relationships that could be construed as a potential conflict of interest.

## Publisher's note

All claims expressed in this article are solely those of the authors and do not necessarily represent those of their affiliated organizations, or those of the publisher, the editors and the reviewers. Any product that may be evaluated in this article, or claim that may be made by its manufacturer, is not guaranteed or endorsed by the publisher.

## Abbreviations

HBV: Hepatitis B Virus
HCV: Hepatitis B Virus
HIV: Human Immunodeficiency Virus
NOS: Newcastle-Ottawa Scale
NSI: Needle-Stick Injury
PRISMA: Preferred Reporting Items for Systematic Reviews and Meta-Analyses.
